# PGC-1s in the Spotlight with Parkinson’s Disease

**DOI:** 10.3390/ijms22073487

**Published:** 2021-03-28

**Authors:** Elena Piccinin, Anna Maria Sardanelli, Peter Seibel, Antonio Moschetta, Tiziana Cocco, Gaetano Villani

**Affiliations:** 1 Department of Basic Medical Sciences, Neuroscience and Sense Organs, University of Bari “Aldo Moro”, 70124 Bari, Italy; elena.piccinin@uniba.it (E.P.); annamaria.sardanelli@uniba.it (A.M.S.); 2 Department of Medicine, University Campus Bio-Medico of Rome, 00128 Rome, Italy; 3 Molecular Cell Therapy, BBZ, Medical Faculty, University of Leipzig, Deutscher Platz 5, 04103 Leipzig, Germany; peter.seibel@bbz.uni-leipzig.de; 4 Department of Interdisciplinary Medicine, University of Bari “Aldo Moro”, 70124 Bari, Italy; antonio.moschetta@uniba.it

**Keywords:** PGC-1, coactivators, neurodegenerative disease, Parkinson’s disease, mitochondria

## Abstract

Parkinson’s disease is one of the most common neurodegenerative disorders worldwide, characterized by a progressive loss of dopaminergic neurons mainly localized in the *substantia nigra pars compacta*. In recent years, the detailed analyses of both genetic and idiopathic forms of the disease have led to a better understanding of the molecular and cellular pathways involved in PD, pointing to the centrality of mitochondrial dysfunctions in the pathogenic process. Failure of mitochondrial quality control is now considered a hallmark of the disease. The peroxisome proliferator-activated receptor gamma coactivator 1 (PGC-1) family acts as a master regulator of mitochondrial biogenesis. Therefore, keeping PGC-1 level in a proper range is fundamental to guarantee functional neurons. Here we review the major findings that tightly bond PD and PGC-1s, raising important points that might lead to future investigations.

## 1. Introduction

Parkinson’s disease (PD) is a neurodegenerative disorder, with the fastest-growing incidence worldwide, causing progressive disability that can be slowed, but not halted, by treatments. The overall number of people affected by this disorder has more than doubled in the last 30 years [1], due not only to the aging of the population, but also to an intensified exposition to industrial chemicals and pollutants, which have been associated with an increased risk of developing the disease [2]. More than 90% of PD cases are sporadic. A small percentage, however, is due to mutations in specific genes.

The first evidence of a tight association between PD and mitochondria was made in 1983 [3]. Since then, researchers have focused on disentangling the intricate network that underlies mitochondrial functions and the onset of the disease [4]. 

Mitochondria are essential organelles in eukaryotic cells that regulate several aspects of energy and lipid metabolism, as well as ion homeostasis and cellular apoptosis. Nonetheless, mitochondria are also the major source of reactive oxygen species (ROS). To assure all these aspects, mitochondria have to work properly, since also minimal modifications compromising mitochondrial functions can have deleterious effects bearing upon the development of a plethora of pathological states, including neurodegenerative diseases. Therefore, mitochondria have evolved several quality control mechanisms that allow the degradation and replenishment of dysfunctional organelles in order to guarantee the fulfilment of all their roles [5]. A correct mitochondrial turnover is of utmost importance in postmitotic cells with obligate aerobic energy metabolism, such as neurons.

Mitochondrial biogenesis is one of the aspects of mitochondrial turnover associated with PD that has been intensely analysed until now. Specifically, studies have been focused on the peroxisome proliferator-activated receptor gamma coactivator 1 (PGC-1) family, long considered as master regulators of mitochondrial biogenesis as well as of antioxidant response. Notably, other neurodegenerative diseases, including Alzheimer’s disease and Huntington’s disease, have been linked to perturbations of PGC-1 activity [6,7].

Here, after briefly recapitulating the major mitochondrial alterations occurring in PD, we summarize much of the work that supports our understanding of the peculiar role played by PGC-1s and how this impacts on neurodegeneration and development of PD.

## 2. Parkinson’s Disease: An Overview

PD is characterized by the loss of pigmented dopaminergic (DA) neurons of the *substantia nigra pars compacta* (SNpc), leading to a depletion of dopamine inputs in the striatum, i.e., the brain nucleus designated to the control of voluntary movements. This dopamine deficit causes the motor symptoms associated with the disease, including bradykinesia, muscle rigidity, and tremors, finally evolving in akinesia, dementia, and death [8]. Lewy bodies (LB) represent another important pathological hallmark of PD. Although the role of LB in PD pathogenesis is still to be defined, it is now clear that they are formed by progressive intracellular aggregation of α-synuclein interspersed in a matrix of lipids, lysosomal structures, and mitochondria [9,10]. Notably, the accumulation of α-synuclein causes severe cellular toxicity, mainly due to disruption of vesicle trafficking and reduced lysosomal degradation capacity [11,12,13,14]. Moreover, the α-synuclein aggregates preferentially bind to mitochondria and cause membrane depolarization and impaired cellular respiration [15]. Recently, a dysregulated lipid profile has been pointed out in fibroblasts isolated from PD patients compared to those from healthy subjects [16,17]. Specifically, an excess of a specific class of lipids, the monounsaturated fatty acids (MUFAs), can exacerbate the α-synuclein pathology leading to trafficking defects [18]. By contrast, saturated fatty acids (SFAs) seem to protect neurons from α-synuclein toxicity [19]. In humans, the stearoyl-CoA desaturase (SCD) is the rate limiting enzyme for the conversion of SFAs to MUFAs, specifically driving the synthesis of oleic and palmitoleic acid [20,21]. In several animal models and human-derived neurons, the inhibition of SCD limits the endogenously produced oleic acid and prevents the DA degeneration via the inhibition of α-synuclein inclusion [19,22]. 

### 2.1. Parkinson’s Disease and Mitochondria: A Tight Bond Underlying Pathogenic Mechanism

Until now, two different kinds of PD have been described, i.e., the familial and the sporadic form, respectively. Although clinically indistinguishable, the latter comprises more than 90% of the cases and has a multifactorial aetiology. Contrarily, the familial form accounts for about 10% of all PD patients and is associated with autosomal and recessive monogenic mutations [23,24,25,26]. However, functional analysis of the mutated genes in familial PD has shed light on the underlying mechanisms that lead to neurodegeneration. Furthermore, genome-wide association studies (GWAS) have provided useful information that closely links the familial and the sporadic forms, since polymorphisms in familial PD genes have resulted as risk factors for the development of sporadic PD [27,28,29].

The first insight on mitochondrial dysfunction in PD dates back more than 30 years, when it was observed that the administration of the mitochondrial toxin 1-methyl-4-phenyl-1,2,3,6-tetrahydropyridine (MPTP) was able to inhibit the respiratory chain complex I, thus inducing DA neuron loss and Parkinsonism [30]. Indeed, complex I represents a sensitive target in neuronal bioenergetic metabolism, due to its controlling role in mitochondrial oxidative phosphorylation (OXPHOS) efficiency and capacity, especially during aging [31,32,33]. Thereafter, numerous other compounds have been utilized in animal models to induce motor symptoms of PD, mainly via the inhibition of the respiratory chain complexes with the consequent generation of oxidative stress and impaired calcium homeostasis which cause cell death in the DA neuronal population [34,35]. 

**Figure 1:**
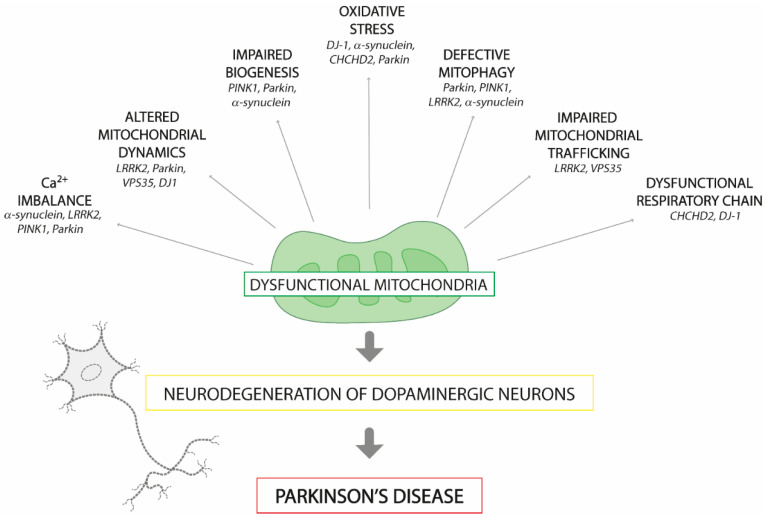
Mitochondrial dysfunctions associated with Parkinson’s disease pathogenesis. The disruption of a gene (in italic) involved in mitochondrial homeostasis may result in the generation of dysfunctional mitochondria in the *substantia nigra pars compacta*, leading to the loss of pigmented dopaminergic neurons. The consequent dopamine deficit causes the motor symptoms associated with the disease. Abbreviations: PTEN-induced putative kinase 1 (*PINK1*), α-synuclein (*SNCA*), leucine-rich repeat kinase 2 (*LRRK2*), vacuolar protein sorting-associated protein 35 (*VPS35*), coiled-coil-helix-coiled-coil-helix domain containing 2 (*CHCHD2*).

Subsequent studies have highlighted a complex interplay between different mitochondrial dysfunctions underlying PD pathogenesis, including impaired biogenesis, defective mitophagy, altered mitochondrial dynamics, and compromised trafficking (Figure 1). These malfunctions are possibly related to the failure of the mechanisms that regulate mitochondrial quality control, leading to electron transport chain dysfunction and a consequent increase in ROS production [36]. Furthermore, accumulation of somatic mutations in mitochondrial DNA (mtDNA) has also been found in PD patients [37].

Several pieces of evidence suggest that PD-associated genes are strictly connected with mitochondrial integrity. Among all the genes identified as monogenic causes of familial PD, Parkin (*PRKN*), PTEN-induced putative kinase 1 (*PINK1*), α-synuclein (*SNCA*), leucine-rich repeat kinase 2 (*LRRK2*), vacuolar protein sorting-associated protein 35 (*VPS35*), *DJ-1*, and coiled-coil-helix-coiled-coil-helix domain containing 2 (*CHCHD2*) are those with a peculiar role in mitochondrial homeostasis.

#### 2.1.1. mtDNA: A Neuronal Clock for Parkinson’s Disease?

Early insights for mtDNA involvement in PD came from the analyses of transmitochondrial cybrids, i.e., cells derived from the fusion of mtDNA-depleted (Rho0, ρ0) cells with platelets from PD patients or controls, respectively, as mtDNA donors [38,39]. Further studies have demonstrated that somatic mtDNA mutations, as well as changes in mtDNA copy number, can be distinguished in PD brain tissues. Indeed, PD subjects display a high level of deleted mtDNA that results in respiratory chain deficiency and the possible formation of ROS [40,41]. Particularly, mtDNA deletions are differently distributed between cytochrome c oxidase (COX)-deficient and COX-positive neurons, with the higher extent in COX-deficient ones, suggesting that mtDNA defects can contribute to the disruption of the respiratory chain and compromise mitochondrial functions, finally playing an important role in DA neurons loss [42]. Notably, most of these mutations are age-related, thus underlining ageing as one of the risk factors for the development of neurodegenerative diseases [43]. A longitudinal study conducted in mice revealed that there is a progressive significant reduction of genes strictly associated with mitochondrial biogenesis and antioxidant response, which is accompanied by a decline in cognitive performance during aging [44].

Additionally, mtDNA depletion can be considered as a possible factor predisposing to PD onset. However, whether a low mtDNA copy number positively or negatively correlates with longevity is still debated. With ageing, normal subjects usually display an increase in the mtDNA levels in *substantia nigra* neurons and a decline in mtDNA in peripheral blood cells [41,45]. Nevertheless, lower mtDNA copy numbers have been observed in the *substantia nigra* of PD patients compared to controls [41,46,47]. Notably, this reduction is preserved also in the peripheral blood and cerebrospinal fluid, therefore representing an interesting biomarker for PD detection [48,49].

#### 2.1.2. PINK1 and Parkin: Two Players, One Axis

*PINK1* and *Parkin* were among the first genes whose mutations were unequivocally linked to the familial autosomal recessive form of PD [50,51]. The PINK1/Parkin pathway plays a crucial role in the mitochondrial quality control, mainly affecting the degradation of damaged mitochondria and the formation of novel organelles. Parkin is an E3 ubiquitin ligase that catalyses the ubiquitination of target proteins for signaling or proteasomal degradation, and it is usually present as a cytosolic auto-inhibited enzyme [52]. To be fully active, Parkin needs to be phosphorylated by PINK1, a serine/threonine-kinase. Under basal conditions, PINK1 is imported through the mitochondrial translocases of the outer and inner membrane (TOM and TIM, respectively), and it is targeted for proteasomal degradation thereafter [53]. Thus, the levels of PINK1 are generally low in healthy mitochondria. However, upon mitochondrial membrane depolarization resulting from acute or prolonged damage, PINK1 is stabilized in the outer mitochondrial membrane (OMM), where it mediates the activation of Parkin by a two-fold mechanism. PINK1 directly phosphorylates Parkin serine 65 (Ser^65^) within its N-terminal ubiquitin-like (Ubl) domain as well as the Ser65 residue of ubiquitin, and the phosphorylation of both of these residues is required for maximal activation of Parkin E3 ligase activity to promote ubiquitin chain assembly [54,55,56,57,58]. Once fully activated, Parkin ubiquitinates OMM proteins for their subsequent proteasomal degradation. Notably, the Parkin-pSer65Ub interaction greatly increases the PINK1 phosphorylation rate of Parkin, thus establishing a feedforward mechanism to amplify the signal [59]. Ubiquitylation of mitochondrial proteins by Parkin is finally decoded by the autophagy machinery that captures the damaged organelles, by assembling an autophagosomal membrane around ubiquitinated mitochondria, and drives their degradation via lysosomal fusion. Remarkably, PINK1 recruits also autophagy receptors optineurin (OPTN) and nuclear dot protein 52 kDa (NDP52) in a Parkin-independent way [60,61], and it is able to negatively regulate mitophagy via TUFm [62]. Although the PINK1/TUFm role may appear quite contradictory, their antagonizing activity may represent a safeguard mechanism to tightly regulate homeostasis, preventing excessive mitophagy or counteracting false positive activation of mitochondrial degradation. Moreover, several enzymes (deubiquitylases, pSer65Ub phosphatases) antagonize the PINK1/Parkin-induced mitophagy. Different translational and post-translational regulators of PINK1 and Parkin have also been recently recognized [63], hence leading to a more detailed mapping of mitophagy pathway in the homeostatic quality control of mitochondria. Intriguingly, mutations in the *Parkin* gene so far identified are characterized by a local change that may propagate to the entire molecule and affect the enzyme stability and its recruitment to damaged mitochondria, thus leading to the accumulation of dysfunctional mitochondria [59,64]. 

In addition to their role in mitophagy, PINK1 and Parkin have been implied also in the maintenance of a functional mitochondrial pool by modulating mitochondrial biogenesis, as discussed in the last part of this manuscript. Although this double role of the PINK1/Parkin pathway in mitochondrial homeostasis has been recently clarified, the physiological stimuli that distinctively activate the destruction or the repair processes have still to be determined. Noteworthy, defects in basal mitophagy were not observed in animal models lacking PINK1 or Parkin, where the loss of either the kinase or the ubiquitin ligase activity is not associated with an increase of mitochondrial mass, despite the injury to DA neurons [36,65,66,67]. This may suggest that other processes rather than mitophagy itself are of pathological relevance for the onset of PD.

#### 2.1.3. α-synuclein: Not Just A Simple Aggregation

Besides *PINK1* and *Parkin*, other PD-associated genes may have a potential role in the mitochondrial quality control. The α-synuclein gene encodes for a protein which forms pathological aggregates that bind to mitochondria and induce organelle permeabilization and mitochondrial membrane depolarization [15,68]. In this view, α-synuclein accumulation increases mitochondrial fragmentation and autophagic lysosomal pathway activation [69]. Moreover, α-synuclein overexpression in the mouse nervous system increases oxidative stress that overwhelms antioxidant defense, as indicated by the extent of lipid peroxidation and the concomitant changes in the level of peroxiredoxin 2, and makes DA neurons more vulnerable [70]. Importantly, mutated α-synuclein neurons under oxidative stress conditions show decreased mitochondrial biogenesis and increased apoptotic cell death due to the disruption of the myocyte enhancer factor 2C (MEF2C)/PGC-1α pathway [71]. Despite all these pieces of evidence, understanding how the mitochondrial dysfunctions caused by α-synuclein underlie PD aetiology is still a matter of debate.

#### 2.1.4. LRRK2: One Protein, Different Functions

Autosomal dominant mutations in *LRRK2* are one of the most common monogenic forms of Parkinson’s disease (PD) accounting for 1–2% of all PD patients. In human-induced pluripotent stem cell (iPSC)-derived DA neurons, LRRK2 forms a complex with the OMM adaptor protein MIRO to promote its removal [72]. MIRO is necessary to anchor mitochondria to microtubule motor complexes and it is usually removed to stop mitochondrial motility [73]. Pathogenic *LRRK2* mutations disrupt MIRO removal and result in a stalling of dysfunctional mitochondria, consequently slowing down organelle turnover [72]. Notably, these results have been confirmed in neurons isolated from sporadic PD patients [72], hence pointing to a crucial role of mitophagy also in idiopathic PD pathogenesis. Furthermore, the increase of LRRK2 kinase activity, due to overexpression of the wild type form or to the mutant G2019S form, alters the interaction between Parkin and Drp1, a dynamin-related protein involved in mitochondrial division [74]. Concomitantly, mutated LRRK2 (G2019S and R1441C) inhibits the interaction between RAB10 and optineurin (OPTN), thus reducing the mitophagy rate [75]. RAB10 is an LRRK2 substrate that accumulates on depolarized mitochondria in a PINK1/Parkin-dependent manner, where it facilitates the binding to OPTN to promote mitophagy [75]. Altogether, these pieces of evidence point to a complex role of LRRK2 in mitochondria trafficking, dynamics, and autophagy.

#### 2.1.5. VPS35: The Mitochondria Traffic Warden

Vacuolar protein sorting 35 (VPS35) is a core component of the retromer trimer required for endosomal membrane-associated protein trafficking. The discovery of dominantly-inherited, late-onset Parkinsonism has recently been linked to a missense mutation in *VPS35* which implicates retromer dysfunction in the pathogenesis of PD [76,77]. VPS35 regulates retrograde delivery from endosomes to the trans-Golgi network and to the plasma membrane, and it is involved in the formation of mitochondria-derived vesicles that shuttle cargo from mitochondria to peroxisomes and lysosomes, thus playing a role in the maintenance of mitochondrial homeostasis and dynamics [78]. VPS35 can recognize and interact with Drp1, thus mediating the removal of Drp1 from mitochondria and its transport via mitochondria-derived vesicles to lysosomes for degradation [79]. When *VPS35* is mutated, or in the presence of oxidative stress, the interaction and consequent degradation of Drp1 becomes more prominent, resulting in an unbalanced regulation of mitochondrial fusion/fission towards the former, which finally leads to mitochondrial dysfunction and neuronal loss [80,81]. Importantly, also the other genes described in the previous chapters may play a role in Drp1 functions: PINK1/Parkin pathway promotes its ubiquitylation and degradation, whereas LRRK2 contributes to its recruitment on mitochondria [82,83,84]. 

#### 2.1.6. DJ-1: A Shield Against Oxidative Stress

DJ-1 belongs to the peptidase C56 protein family and is encoded by the *PARK7* gene, whose mutations have been associated with autosomal recessive early onset PD [85]. Ubiquitously expressed, the functions of this protein are still not well understood, although the generation of a DJ-1 deficient mouse model confirmed its involvement in PD pathogenesis [86]. Indeed, knocking down DJ-1 produces alterations in mitochondrial morphology and dynamics [87,88,89]. Notably, flies lacking DJ-1 also manifest dysfunctions close to those observed in *Parkin* or *PINK1* mutants [90]. Curiously, DJ-1 upregulation can rescue the phenotype in PINK1 *null* animals, thus indicating the existence of simultaneous pathways controlling mitochondrial turnover [90].

To fully exert its neuroprotective role, DJ-1 has to translocate from the cytosol to the mitochondria, which usually occurs in response to mitochondrial stress [91,92]. Specifically, DJ-1 expression confers protection against oxidative stress via the activation of different redox-sensitive transcription programs and through the interaction and stabilization of ROS-scavenging enzymes as well as of several proteins linked to proteolysis, SUMOylation, and cell death [93,94,95]. Recently, it has been discovered that DJ-1 is also essential to regulate ATP synthase function and mitochondrial membrane permeability, protecting against cell death and enhancing neuronal processes [96].

#### 2.1.7. CHCHD2: In Close Connection with Mitochondrial Electron Transport Chain Complex IV

Mutations of the *CHCHD2* gene have been found in autosomal PD [97]. CHCHD2 is a mitochondrial intermembrane space protein with a significant role in maintaining the integrity of mitochondrial cristae. Principally, CHCHD2 binds to and modulates the activity of the electron transport chain complex IV [98]. Under stress conditions, CHCHD2 translocates to the nucleus where it regulates the transcription of complex IV subunits [98]. Mutations affecting *CHCHD2* alter cytochrome c and complex IV activity, therefore resulting in impaired respiration and exacerbating ROS generation that altogether contribute to DA neuron loss [99]. It is worth noting that even mild decreases of complex IV activity can have a dramatic impact on neuronal bioenergetic functions due to the tight control exerted by the terminal enzyme of the electron transport chain on respiratory fluxes and, consequently, on mitochondrial membrane potential [100,101,102]. Although several pieces of evidence have been obtained connecting CHCHD2 to mitochondrial dysfunction, its role in sporadic forms of PD has still to be clarified.

## 3. PGC-1 Family: The Masters of Mitochondrial Biogenesis

The family of peroxisome proliferator-activated receptor γ coactivator 1 comprises three members, PGC-1α, PGC-1β, and PGC-related coactivator (PRC). PGC-1s act as ‘molecular switches’ in many metabolic pathways, coordinating transcriptional programs involved in the control of cellular metabolism and overall energy homeostasis as well as antioxidant defence [103]. Their versatile actions are achieved by interacting with different transcription factors and nuclear receptors in a tissue-specific manner.

Notably, in analysing the *PGC-1α* gene, it has emerged that different variants originating from diverse transcription start sites exist [104]. This has led to the identification of a so-called “brain variant” that is more abundant in the human brain than the canonical isoform [105]. Moreover, an alternative splicing event, which introduces a premature stop codon, yields a shortened version of the coactivator, named *NT-PGC-1α* that is highly abundant in the mouse brain [106]. 

### 3.1. PGC-1s’ Architecture

The modular structure of PGC-1s is highly conserved among all three members of the family. The N-terminus of PGC-1 contains a strong transcriptional activation domain, whereas the C-terminal region holds a serine/arginine rich domain and an RNA binding domain that couples pre-mRNA splicing with transcription [107]. At its N-terminus domain, PGC-1 interacts with several histone acetyltransferase (HAT) complexes, including cAMP response element-binding protein (CREB)-binding protein, p300, and steroid receptor coactivator-1 (SRC-1) [108]. On the other side, in the C-terminal region, other activation complexes dock PGC-1, including the TRAP/DRIP (thyroid receptor-associated protein/vitamin D receptor-interacting protein), also known as Mediator complex, which facilitates direct interaction with the transcription initiation machinery [109], and the SWI/SNF (switch/sucrose non-fermentable) that acts as a chromatin-remodelling complex, through its interaction with BAF60a [110]. This peculiarity of PGC-1s to function as a protein docking platform for the recruitment and assembly of various chromatin remodelling and histone-modifying enzymes, which easily allow the access of the transcription machinery to DNA by altering the local chromatin state, contributes to the remarkably powerful PGC-1s coactivation capacity.

Furthermore, an alternative mechanism to increase gene expression relies on the capability of the PGC-1α transcriptional activator complex to displace repressor proteins, such as histone deacetylase and small heterodimer partner (SHP), on its target promoters [111].

PGC-1α and PGC-1β share a common similar domain in the internal region, which functions as a repression domain [108], and several clusters of conserved amino acids, such as the LXXLL motif that is recognized by nuclear receptors and host cell factor 1 interacting motif [112,113]. Although it contains the same activation domain and RNA-binding domain as the other members of the family, PRC shows poor sequence similarity to PGC-1α and PGC-1β [114]. Few studies have been conducted until now to elucidate the functions of PRC, and to our knowledge, none of them focus primarily on its activity in the brain. Therefore, this member of the PGC-1 family will be not considered in this review.

### 3.2. PGC-1s’ Activity: Boosting Mitochondrial Functions

Although PGC-1s display an extremely powerful autonomous transcriptional activity, the mechanism through which PGC-1s activate gene expression is to date poorly understood. The spatial and temporal assemblance of the several activation complexes to PGC-1 is still unknown. The major current hypothesis is that PGC-1 binds to a specific transcription factor in the promoter region, followed by the recruitment of P300 and TRAP/DRIP complexes which opens the chromatin through histone acetylation activity, thus allowing the initiation of the transcription via RNA polymerase II (RNApolII). Moreover, the involvement of additional proteins in RNA elongation and processing as part of the PGC-1 complexes suggests that it might move along the elongating RNA and take part in the mRNA maturation. To terminate gene expression, the acetyltransferase GCN5 acetylates PGC-1 at several lysine residues, inducing re-localization of PGC-1 from the promoter region to subnuclear foci where its transcriptional activity is inhibited [115,116]. On the contrary, sirtuin 1 (SIRT1) activates PGC-1 by deacetylating lysine residues, thus inducing the expression of PGC-1 target genes [117].

Another level of complexity is introduced by other post-translational modifications of PGC-1s, such as phosphorylation and methylation, as well as by the interaction with co-repressors which alters PGC-1s stability and activity. PGC-1α could be directly phosphorylated by three different kinases. p38 MAP kinase and AMP-activated protein kinase (AMPK) phosphorylate PGC-1α, stabilizing the protein and leading to an increase in gene expression activity [118,119,120,121]. Differently, protein kinase B (AKT) produces a more unstable PGC-1α protein, with consequently decreased expression of target genes [122]. Furthermore, PRMT1 (protein arginine N-methyltransferase 1) methylates PGC-1α on three arginine residues in the C-terminus, hence promoting its activation [123].

It is plausible that PGC-1α acts in multiple transcriptional complexes whose composition might depend on the specific target genes as well as on different metabolic signals [124]. Indeed, PGC-1s may interact with different transcription factors, activating diverse biological programs in a tissue-specific manner. PGC-1α was originally described through its functional interaction with peroxisome proliferator-activated receptor γ (PPARγ) in brown adipose tissue (BAT), a mitochondria rich tissue, where it regulates adaptive thermogenesis in response to cold [125]. Further studies revealed that the PGC-1s carry out a plethora of biological responses finalized to manage situations of energy shortage. One of the best characterized functions of PGC-1s resides in their ability to promote mitochondrial biogenesis by coactivating different transcription factors, such as the oestrogen-related receptor α (ERRα), the Nuclear Respiratory Factors 1 and 2 (NRF1 and NRF2, respectively), and the transcriptional repressor protein yin yang 1 (YY1) [126,127,128]. These transcription factors, in turn, regulate the expression of mitochondrial transcription factor A (*TFAM*), which plays an essential role in mtDNA replication, maintenance, and transcription [129]. Moreover, both NRF1 and NRF2 guarantee mitochondrial homeostasis by modulating the expression of nuclear genes for components of the OXPHOS system, such as ATP synthase, cytochrome c, and cytochrome c oxidase [130,131]. In addition to the powerful activity as master regulators of mitochondrial biogenesis, PGC-1s positively control the expression of genes involved in antioxidant response [132]. Finally, PGC-1s can also regulate several other metabolic pathways in different tissues, including gluconeogenesis, fatty acid β-oxidation, thermogenesis, and *de novo* lipogenesis [103]. The gluconeogenic pathway is mainly initiated by PGC-1α, rather than PGC-1β [133]. Particularly, PGC-1α activates gluconeogenesis interacting with forkhead box protein O1 (FOXO1), hepatocyte nuclear factor 4α (HNF4α), and glucocorticoid receptor (GR) [134,135,136]. The capacity to sustain fatty acid catabolism is particularly evident in the fasted liver, where PGC-1s act as powerful coactivators of peroxisome proliferator-activated receptor α (PPARα) and promote the synthesis of genes involved in fatty acid oxidation, such as medium-chain acyl-CoA dehydrogenase (*MCAD*) and carnitine palmitoyltransferase 1A (*CPT1A*) [137,138]. In BAT, PGC-1s are able to drive the production of heat by inducing the expression of uncoupling protein 1 (*UCP1*) [139,140]. Finally, PGC-1β alone is able to regulate *de novo* lipogenesis and very low-density lipoprotein trafficking in the liver, mainly by interacting with liver X receptor (LXR) and sterol regulatory element-binding protein 1c (SREBP1c), leading to the expression of fatty acid synthase (*FASN*) and *SCD1* [141,142,143]. It is important to note that in many tissues the functions of PGC-1α and PGC-1β may overlap. However, in some organs like the liver, these two coactivators exert opposite functions, with PGC-1α mainly regulating fatty acid β-oxidation during fasting and PGC-1β activating *de novo* synthesis of fatty acids after the intake of a meal enriched in lipids [144]. Although still not well investigated, the occurrence of the same phenomenon in other organs cannot be excluded.

## 4. PGC-1s in Parkinson’s Disease

The association between PD and alteration of mitochondrial homeostasis has been extensively reported. However, only recently has it been highlighted that disruptions of mitochondrial biogenesis and dynamics, rather than mitophagy, are closely associated with the disease onset. This has resulted in a detailed evaluation of the role of PGC-1s on the onset and progression of PD. 

The first evidence of the involvement of PGC-1s in neurodegenerative diseases came from two independent studies on whole body PGC-1α knock out (PGC-1αKO) mice [137,145]. Morphologically, the absence of PGC-1α in the brain results in a well-preserved cerebral cortex with the presence of vacuoles in neurons containing aggregates of membranous material [137]. Nevertheless, these mice display behavioural abnormalities peculiar to neurological disorders, indicative of lesions in the striatum [145]. A direct demonstration of the link between PGC-1α and PD was the higher vulnerability of PGC-1αKO mice to the neurodegenerative effects of MPTP and kainic acid, due to the lack of the PGC1α-dependent induction of the antioxidant response [146]. Furthermore, cultured PGC1α-*null* nigral neurons were more sensitive to the accumulation of the overexpressed human α-synuclein [147] and conditional PGC1α-KO in the *substantia nigra* of adult mice caused DA neuron loss associated with a marked decline of mitochondrial biogenesis protein markers [148]. Indeed, while the expression of this coactivator protects neuronal cells from ROS-induced cell death via induction of several detoxifying enzymes (superoxide dismutase 2, SOD2; glutathione peroxidase 1, GPX1), its ablation results in the accumulation of nitrotyrosylated proteins and loss of DA neurons [146]. Furthermore, transgenic mice in which the expression of the PGC-1s target gene TFAM has been disrupted display lower mtDNA expression and respiratory chain deficiency that result in the adult onset of Parkinsonism [149]. All these insights provide the impetus to deepen the knowledge of PGC-1s in PD.

Despite PGC-1α and PGC-1β are both powerful coactivators of mitochondrial biogenesis and antioxidant response, PGC-1α has been far more investigated as compared to PGC-1β, especially in the context of PD. Although PGC-1α and PGC-1β control mitochondrial capacity in an additive and independent manner in different subtypes of neurons, the overexpression of PGC-1α significantly reduces the PGC-1β level [147,150]. Furthermore, while PGC-1α can compensate PGC-1β loss and restore the induction of antioxidant genes, PGC-1β fails to cope with the absence of PGC-1α, being only slightly induced in PGC-1α *null* mice [146,147]. However, since in other tissues PGC-1β is able to drive genes involved in *de novo* lipogenesis, including *SCD1* [141,142], and given that *SCD1* expression has proved to be deleterious for PD, it would be intriguing to evaluate if PGC-1β retains this ability also in DA neurons and whether the activation of this pathway may have detrimental effects. 

### 4.1. Deepening the Role of PGC-1α in Parkinson’s Disease

Numerous studies have been carried out to fully elucidate the role played by PGC-1α in PD. A comparative analysis of a large cohort of PD patients and age-matched controls has revealed that two PGC-1α variants are associated with the risk of PD onset (rs6821591 CC and rs2970848 GG) [151]. Further studies have led to the identification of different PGC-1α isoforms in the brain, including a truncated 17 kDa protein that lacks the LXXLL site of interaction with several transcription factors [148,152]. This 17 kDa isoform has been found upregulated in the *substantia nigra* of PD patients, where it inhibits the coactivation of several transcription factors by the full-length PGC-1α [152]. The selective knockout of different *PGC-1α* isoforms in mice may lead to a decrease of dopamine content in the striatum and to an associated loss of DA neurons [148]. Accordingly, in human PD tissues, the levels of PGC-1α and of mitochondrial markers are reduced compared to control patients and are negatively correlated with the severity of the disease [148,153,154,155,156,157]. Conversely, primary fibroblasts from PD patients display upregulation of PGC-1α, even if its target genes involved in mitochondrial biogenesis and fatty acid β-oxidation processes are unchanged or downregulated and mitochondria display significant morphometric changes [158,159]. This may suggest that a post-translational mechanism may also occur, thus jeopardizing the elevated quantity of the coactivator and interfering with its activity.

The low expression of PGC-1α observed in the PD brain is probably due to the high level of gene methylation that has been found in PD patients [155]. Dense DNA methylation is usually associated with gene repression, and the *PGC-1α* promoter is proximal to a non-canonical cytosine methylation site that is epigenetically modified in the brain of sporadic PD patients [153]. Notably, free fatty acids can induce the hypermethylation of the *PGC-1α* promoter. The administration of palmitate to cortical neurons *in vitro* and to a mouse model of PD *in vivo* causes promoter hypermethylation, thus lowering the level of PGC-1α and mitochondria-associated genes as well as the concomitant induction of pro-inflammatory genes [153]. Curiously, both a population-based case-control study and a prospective study indicate that a higher caloric intake, due to elevated consumption of animal-derived saturated fatty acids, tends to be associated with a greater risk of PD [160,161]. Moreover, rats subjected to high-fat diet feeding and to infusion of 6-hydroxydopamine displayed DA neurons depletion in the striatum, despite the absence of differences in locomotor activity [162]. Altogether this suggests that a high dietary introit of fatty acids, especially saturated ones, may repress the expression of *PGC-1α* and related mitochondrial genes, thus lowering the threshold for developing PD. 

Studies performed in *Drosophila melanogaster* further corroborate the association between disrupted PGC-1α functions and PD onset. To some extent, the high degree of conservation between *PGC-1α* and its *D. melanogaster* homolog *Spargel* and the lack of gene redundancy make this organism an ideal model system to determine the role of PGC-1α in PD. The inhibition of Spargel via RNAi in DA neurons causes an altered climbing activity, with an unexpected increase of mean lifespan probably ascribable to the mitochondrial unfolded protein response and/or a ROS-dependent mitohormesis [163]. Further studies have demonstrated that silencing Spargel in flies increases the PD related phenotypes, including climbing defects, decreased mitochondrial mass, and lower dopamine levels [164]. However, the genetic or pharmacological activation of Spargel is sufficient to rescue the disease phenotype [164]. 

Coherently, PGC-1α *null* mice display abnormal mitochondria in neurons and are more prone to oxidative stress that may be eventually related to neurodegeneration. Nonetheless, re-expression of PGC-1α in these mice restores mitochondrial functions and oxidative stress detoxification [147].

The overexpression of PGC-1α in several *in vitro* and *in vivo* models results in an overall protection against neurodegeneration. Treatment of the SH-SY5Y neuroblastoma human cell line with the neurotoxin N-methyl-4-phenylpyridinium leads to serious mitochondrial damage that can be functionally reversed by the overexpression of PGC-1α [165]. Moreover, resveratrol treatment in Parkin-mutated fibroblasts promotes PGC-1α activity via SIRT-1, thus resulting in increased mitochondrial biogenesis together with lower ROS accumulation that together ameliorate the phenotypic impact of the mitochondrial dysfunctions caused by the Parkin mutation [166]. Accordingly, transgenic mice overexpressing PGC-1α in MPTP-treated DA neurons display induction of the antioxidant genes *Sod2* and thioredoxin 2 (*Trx2*) as well as increased OXPHOS, which collectively promote neuronal viability and prevent striatal dopamine loss [167]. Conversely, the adenovirus-mediated overexpression of PGC-1α in the *substantia nigra* of mice increases their susceptibility to MPTP, as indicated by the loss of DA neurons [168]. This deleterious effect may be ascribable to the high level of PGC-1α activity caused by the viral vector microinjection and may shed light on the importance of a balanced regulation of this coactivator to achieve beneficial effects.

### 4.2. PGC-1α and Parkinson’s Mutated Genes: Defining The Network Implicated in Parkinson’s Disease

**Figure 2:**
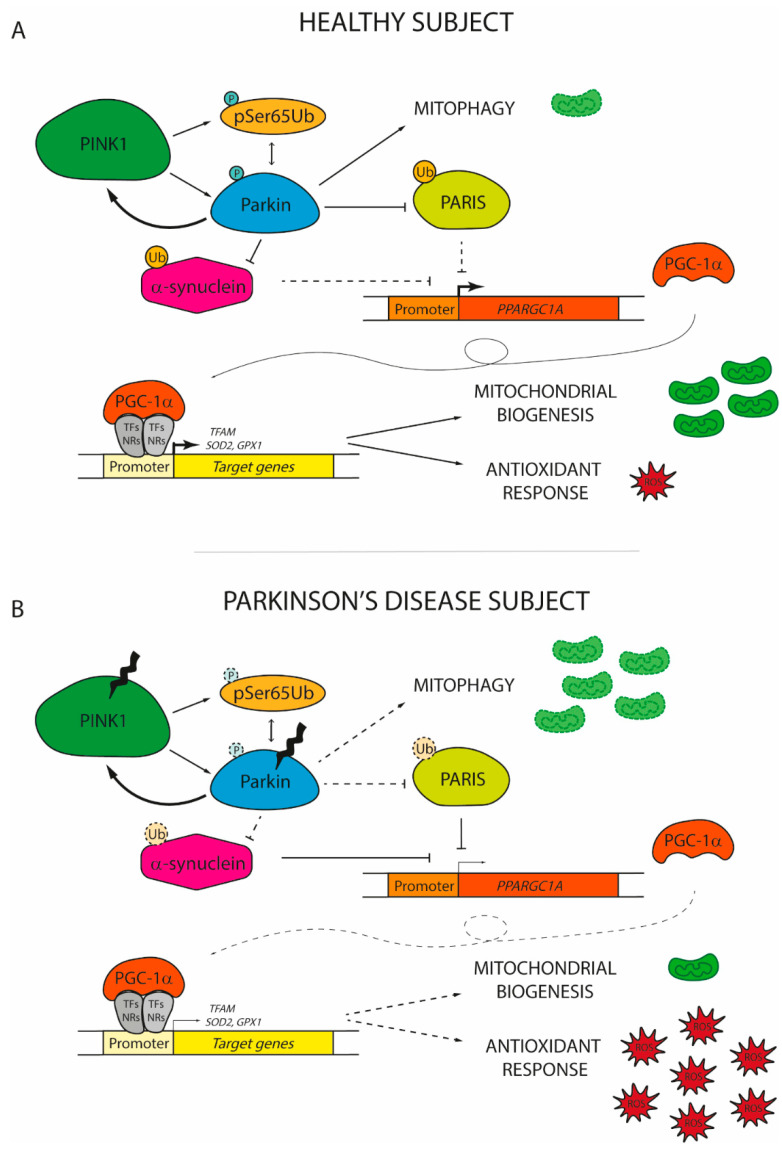
The role of peroxisome proliferator-activated receptor gamma coactivator 1 (PGC-1α) in the onset of Parkinson’s disease. (**A**) In healthy subjects, damaged mitochondria are promptly replaced with new functional organelles. Mitochondrial depolarization induces PTEN-induced putative kinase 1 (PINK1) to phosphorylate serine 65 residue of ubiquitin (pSer65Ub) and of Parkin, which in turn interact together to increase the PINK1 phosphorylation rate of Parkin. The activation of the PINK1/Parkin pathway promotes the degradation of dysfunctional mitochondria via mitophagy and concomitantly induces the expression of PGC-1α by the ubiquitination of PARIS and α-synuclein. By coactivating nuclear receptors (NRs) or transcription factors (TFs), PGC-1α promotes the expression of genes involved in mitochondrial biogenesis (mitochondrial transcription factor A, *TFAM*) and antioxidant response (superoxide dismutase 2, *SOD2*; glutathione peroxidase 1, *GPX1*). (**B**) In Parkinson’s disease individuals, PINK1 and Parkin usually display loss-of-function mutations. Therefore, the PINK1/Parkin axis cannot prevent the accumulation of dysfunctional mitochondria due to its inability to sustain mitophagy. At the same time, altered Parkin fails to promote the degradation of both PARIS and α-synuclein, which start to accumulate in the nucleus inhibiting *PGC-1α* transcription. The low levels of PGC-1α observed in PD patients are not sufficient to induce the expression of genes involved in the renewal of mitochondria and in the antioxidant response. Thereby, reactive oxygen species (ROS) start to accumulate, finally leading to the damage and the death of dopaminergic neurons. Dashed lines and soft colours represent inhibited actions/pathways.

Besides the roles of PGC-1α in different PD scenarios based on its capacity to boost mitochondrial biogenesis and the antioxidant response discussed above, it is now clear that PGC-1α protects against neurodegeneration as a player of a more intricated mechanism whose failure may drive the onset and progression of PD (Figure 2).

The expression of *PGC-1α* is finely regulated by PARIS, a KRAB and zinc finger protein that accumulates in the human PD brain [169]. PARIS acts as a physiological transcriptional repressor of *PGC-1α*, downregulating the coactivator and its target genes, [169]. Generally, the amount of PARIS is tightly controlled by the PINK1/Parkin axis, which mediates PARIS degradation via ubiquitination [170]. However, modifications to either PINK1 or Parkin that alter this regulatory pathway allow PARIS to accumulate inside the neurons [171,172,173]. The overexpression of PARIS negatively affects mitochondrial biogenesis causing progressive DA neuron degeneration and loss [172]. In flies, the ubiquitous expression of PARIS results in shortened lifespan and climbing defects that are promptly reversed by PINK1, Parkin, or PGC-1α overexpression [172]. Noteworthy, the loss-of-function of Parkin in mice and in human-derived cells leads to mitochondrial respiratory function decline coupled with a decrease of mitochondrial mass and of the antioxidant response, a phenotype that closely reminds those of PARIS-overexpressing cells [171,173]. Accordingly, the reduction of PARIS level in Parkin knockout cells and mice is sufficient to restore mitochondrial biogenesis and cellular respiration [171,173]. By contrast, the effect of Parkin on mitochondrial density is abolished in PGC-1α *null* cortical neurons *in vitro* and the synergic action on mitochondrial functions given by the co-expression of both Parkin and PGC-1α provides significant neuroprotection [174]. This indicates that Parkin is fundamental to ensure the proper action of PGC-1α to stimulate mitochondrial biogenesis, by shutting down PARIS.

PINK1/Parkin axis plays a double role in controlling both the genesis and the destruction of mitochondria. Therefore, it is easy to wonder how the PGC-1α action is finely tuned to keep mitochondrial homeostasis and if the activation of mitophagy in response to damage can start a signal that activates PGC-1α in order to restore the mitochondrial pool. Although this is still an open question, it is clear that the rescue of mitophagy in Parkin knockout mice does not increase *PGC-1α* expression and activity [171]. However, PGC-1α regulates the abundance of mitofusin2 (Mfn2), a protein with a central role in mitochondrial fusion, whose loss in nigrostriatal DA neurons in mice leads to a neurodegenerative phenotype [174,175]. Once more, this observation provides clues for the existence of a precise regulatory loop that underlies mitochondrial homeostasis through PGC-1α.

Although no direct interaction has been observed between PGC-1α and DJ-1, since DJ-1 can compensate for PINK1 loss, it will be intriguing to evaluate if PGC-1α functions can be rescued by DJ-1 expression in PD cells in terms of mitochondrial biogenesis and antioxidant response [90,176].

In addition to PINK1 and Parkin, several studies have demonstrated a strong interference with other genes frequently mutated in PD. One of them is α-synuclein, whose overexpression and oligomerization negatively correlates with PGC-1α level in the human PD brain as well as in murine and cell culture models of α-synuclein oligomerization [156]. Particularly, under oxidative stress, α-synuclein may localize in the nucleus where it specifically binds to the *PGC-1α* promoter, decreasing its activity. By reducing the expression of the coactivator and related target genes, α-synuclein impairs the mitochondrial functions [177]. Intriguingly, the ablation of PGC-1α renders both mice and human neurons more prone to α-synuclein accumulation and toxicity [147,178]. By contrast, the pharmacological activation or genetic overexpression of PGC-1α reduces α-synuclein oligomerization and attenuates neurotoxicity *in vitro* [156,177]. The reciprocal influence of PGC-1α and α-synuclein generates a vicious cycle that may play an important role in the disease progression. Since α-synuclein can be ubiquitinated by Parkin [179,180], it would also be interesting to understand how the different actors involved in PD pathogenesis may act in concert to protect from neurodegeneration and neuronal loss.

## 5. Conclusions

Mitochondrial biogenesis is a peculiar aspect underlying PD pathogenesis. Disruptions of several genes closely associated with the disease may lead to the downregulation of PGC-1α, with consequent loss of mitochondria and bioenergetic decline. The restoration of the level of PGC-1α may represent an appealing opportunity for therapeutic treatments. However, a PGC-1α targeted therapy is not so feasible due to the lack of functional ligand- and DNA-binding domains. A possible modulation of this coactivator may be achieved by manipulating enzymes that post-transcriptionally regulate its activity [181]. A recent study pointed out that serotonin may positively affect mitochondrial biogenesis in cortical neurons via PGC-1α [182]. Of note, it is important to tightly control the level of PGC-1α, since indiscriminate changes of its expression in both directions may have deleterious effects. A better understanding of the intricate network subtended to PGC-1α may provide helpful insights into the development of more precise and effective PD therapies.

## Data Availability

Not applicable.
